# Self-Assembled Au Nanoparticle Monolayers on Silicon in Two- and Three-Dimensions for Surface-Enhanced Raman Scattering Sensing

**DOI:** 10.1021/acsanm.2c01904

**Published:** 2022-08-15

**Authors:** Theresa Bartschmid, Amin Farhadi, Maurizio E. Musso, Eric Sidney Aaron Goerlitzer, Nicolas Vogel, Gilles R. Bourret

**Affiliations:** † Department of Chemistry and Physics of Materials, University of Salzburg, Jakob Haringer Strasse 2A, 5020 Salzburg, Austria; ‡ Institute of Particle Technology, Friedrich-Alexander University Erlangen-Nürnberg, Cauerstrasse 4, 91058 Erlangen, Germany

**Keywords:** Au nanoparticles, SERS, plasmonics, silicon nanowires, metal-assisted chemical etching, self-assembly

## Abstract

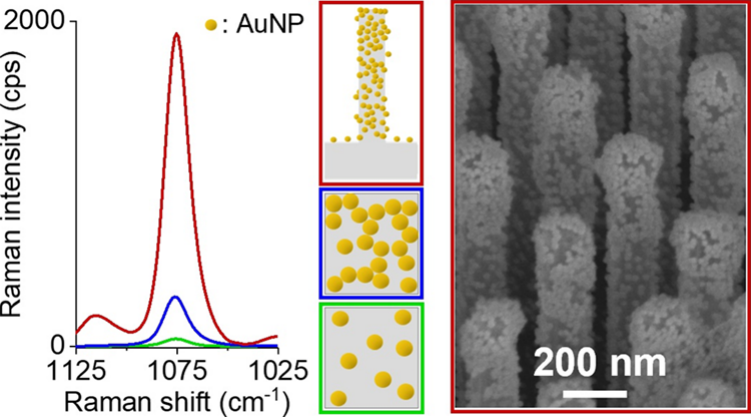

Gold nanoparticle/silicon composites are canonical substrates for sensing applications because of their geometry-dependent physicochemical properties and high sensing activity via surface-enhanced Raman spectroscopy (SERS). The self-assembly of gold nanoparticles (AuNPs) synthesized via wet-chemistry on functionalized flat silicon (Si) and vertically aligned Si nanowire (VA-SiNW) arrays is a simple and cost-effective approach to prepare such substrates. Herein, we report on the critical parameters that influence nanoparticle coverage, aggregation, and assembly sites in two- and three-dimensions to prepare substrates with homogeneous optical properties and SERS activity. We show that the degree of AuNP aggregation on flat Si depends on the silane used for the Si functionalization, while the AuNP coverage can be adjusted by the incubation time in the AuNP solution, both of which directly affect the substrate properties. In particular, we report the reproducible synthesis of nearly touching AuNP chain monolayers where the AuNPs are separated by nanoscale gaps, likely to be formed due to the capillary forces generated during the drying process. Such substrates, when used for SERS sensing, produce a uniform and large enhancement of the Raman signal due to the high density of hot spots that they provide. We also report the controlled self-assembly of AuNPs on VA-SiNW arrays, which can provide even higher Raman signal enhancement. The directed assembly of the AuNPs in specific regions of the SiNWs with a control over NP density and monolayer morphology (i.e., isolated vs nearly touching NPs) is demonstrated, together with its influence on the resulting SERS activity.

## Introduction

Nanostructured surfaces have unique and tunable, size-dependent physicochemical properties that enabled their use in a variety of applications, such as chemical and optical sensing,^1−11^ nanoelectronics,^6,7,12−16^ catalysis,^7,8,17^ solar cells^6,8,18^ or biomedical applications.^6,9,13−15,19^ In that regard, the integration of gold nanoparticles (AuNPs) with silicon (Si) surfaces provides a range of opportunities thanks to the favorable combination of the tunable optoelectronic properties of the AuNPs and Si.^1,5,7,8,20−22^ Because AuNPs@Si substrates are biocompatible, can be selectively functionalized with a wide variety of molecules, and are chemically stable in many different environments, they have become a key model system for the design and study of nanostructured surfaces with tailored properties.^2−5,7,11,12,18−21,23^

AuNPs can sustain localized surface plasmon resonances (LSPRs) that generate large enhancements of the electric-field (E-field) at the NP surface, increasing light absorption and scattering.^24,25^ The E-field enhancement can reach particularly large values within hot spot regions, which are usually NPs in close proximity.^25−29^ Such hot spots are well suited for surface-enhanced Raman spectroscopy (SERS), where the incident and the scattered E-fields are enhanced at the metal NP surface.^26,27,30−33^ Integrating AuNPs with photonic structures, such as vertically aligned Si nanowire (VA-SiNW) arrays, has also emerged as an efficient strategy to enhance the E-field at the AuNP surface without requiring such nanoscale gaps between the NPs,^4,5,8,34−36^ which can be experimentally difficult to achieve in a uniform manner.^4,5,8,26,37^ Indeed, VA-SiNW arrays provide tunable optical properties and can increase the E-field at the Si surface via a variety of optical effects, such as light trapping, waveguiding, diffractive effects, and the excitation of Mie and Fabry-Pérot resonances.^8,17,22,36,38,39^ Thus, decorating SiNWs with AuNPs provides the possibility to couple the LSPR with the E-field generated along the SiNWs,^1,8,36^ further enhancing the E-field at the AuNP surface, while also providing increased NP loading thanks to the three-dimensional geometry of the nanowire array.^4,5,34−36^

Top-down methods, such as electron-beam or nanoimprint lithography, have been successfully used for preparing well-defined Au or other metallic nanostructures on Si.^23,40−43^ They require, however, expensive and elaborated lab equipment and clean-room conditions.^40,41,44,45^ Densely packed AuNP films can be formed via self-assembly at a liquid/liquid interface, which can be later transferred to a planar substrate.^46−48^ However, the postfunctionalization of the AuNPs with a structure-directing molecule, such as mercapto-polyethylene glycol, 1-dodecanethiol, acrylamide molecules, or perfluorodecanethiol, complicates the approach, while the deposition of the resulting AuNP monolayers on substrates with complex topographies, such as VA-SiNW arrays, has not been successfully demonstrated.^46−48^ The self-assembly of presynthesized AuNPs on functionalized Si, on the other hand, offers a low-cost and simple route that is amenable for mass-production and is compatible with the coating of nanostructured silicon.^21,23,44,46−49^ The self-assembly process is self-limited, providing the opportunity to control AuNP coverage, and is potentially compatible with NPs of different sizes, compositions, and shapes.^21,23,50^ However, the preparation of substrates with homogeneous AuNP assemblies providing uniform E-field enhancements over large areas remains a challenge.^23,26^ To prepare such AuNPs@Si substrates, silanes containing amino- or mercapto-groups have been previously used to form siloxane bonds with the Si surface and to direct the assembly of AuNPs at the Si surface either via electrostatic interaction or covalent bonding.^21,51−53^ Both the Si functionalization and the AuNP assembly step are sensitive to the experimental conditions. Among others, the quality of the silane layer is highly affected by the presence of water, temperature, heating speed, reaction time, and silane concentration, as well as the storage conditions after the functionalization itself has been completed.^21,52−54^ The AuNP assembly on the Si surface is also sensitive to the pH and ionic strength of the solution, AuNP size and capping ligand, and immersion time in the AuNP solution.^21,23,51,55^ This self-assembly process is further complicated if topographically structured substrates, such as SiNWs, are to be uniformly coated because geometric restrictions may cause changes in accessibility and concentration profiles of the NPs to be deposited. In particular, the effect of the AuNP monolayer density and morphology (i.e., isolated vs nearly touching NPs) on the resulting optical properties and SERS activity has not been thoroughly addressed to date.

**Figure 1:**
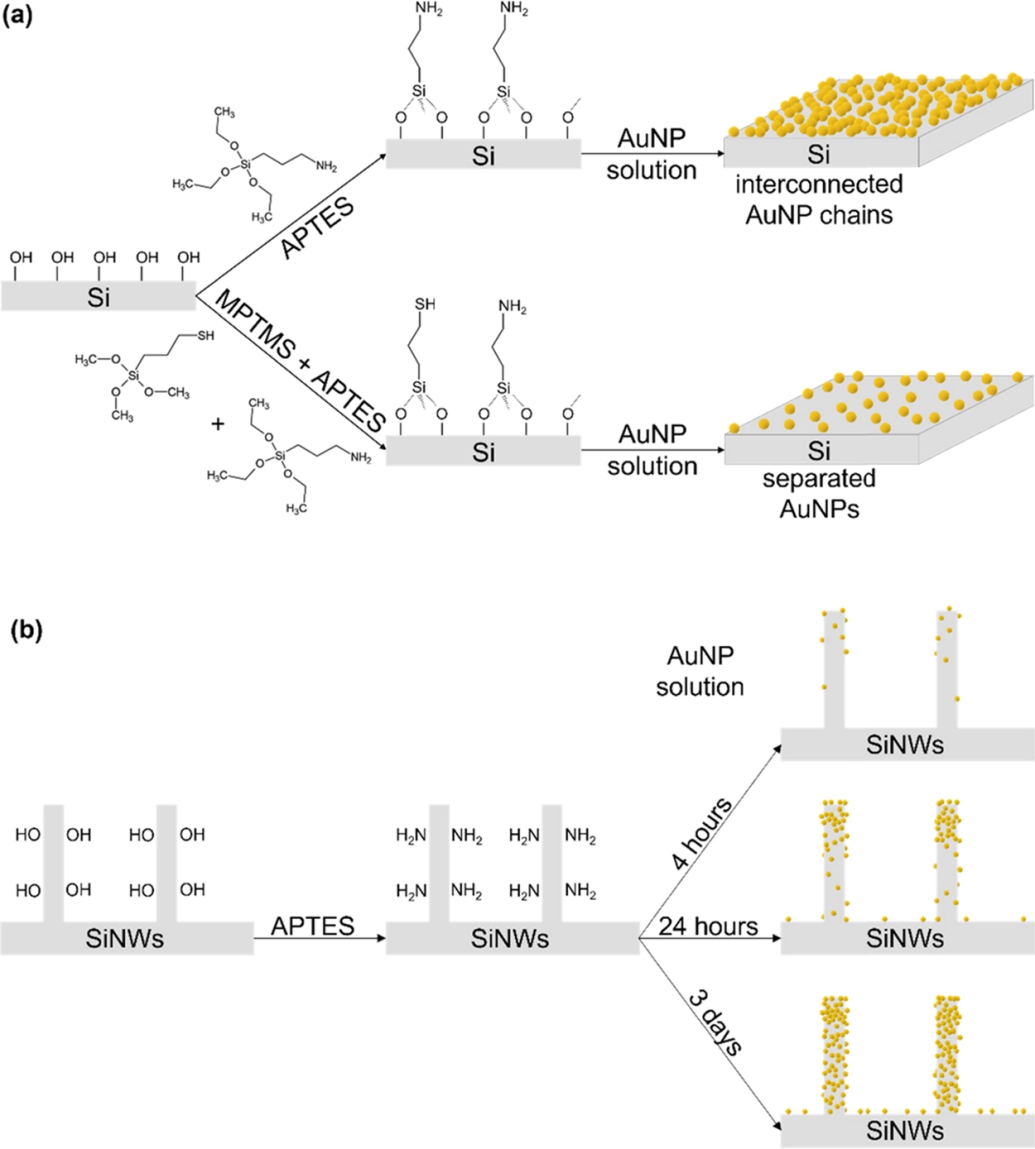
Schematic overview of the AuNP monolayer formation on flat Si and VA-SiNWs. (a) Flat Si: The introduction of hydroxy-groups to the Si surface followed by the condensation reaction with two different silanes (APTES and MPTMS) leads to the functionalization of the Si surface with amino- and mercapto-groups. Subsequent incubation in the AuNP solution, previously synthesized via the Turkevich citrate-route,^57−60^ leads to the homogeneous formation of AuNP monolayers with well-defined aggregation states (isolated vs interconnected AuNPs), depending on the silanes used to functionalize the Si surface. (b) VA-SiNWs functionalized with amino-groups using APTES: Subsequent incubation in the AuNP solution, previously synthesized via the Turkevich citrate-route,^57−60^ for varying times leads to the spatioselective deposition of the AuNPs along the SiNW long axis with tunable surface densities.

Herein, we report a simple technique to control the aggregation, coverage, and assembly sites of negatively charged citrate-protected AuNPs (average diameter of 17 nm) on both flat Si model surfaces and VA-SiNW arrays with a 3D topography. The Si surface was functionalized using (3-aminopropyl)triethoxysilane (APTES) and (3-mercaptopropyl)trimethoxysilane (MPTMS), providing Si surfaces covered with amino- and mercapto-groups. Subsequent incubation of the Si substrates in the AuNP solution leads to the formation of homogeneous AuNP monolayers (Figure 1a). Appropriate synthetic conditions are given to obtain dense monolayers of either well-separated AuNPs or interconnected AuNP chains composed of up to ca. 100–200 NPs in close contact. In particular, a simple route was found to avoid the adsorption of micron-sized extended AuNP aggregates, which occurs during the AuNP self-assembly and significantly limits the homogeneity of the prepared substrates. Additionally, we report on the activity of these substrates for SERS sensing using 4-mercaptobenzoic acid (4-MBA) as analyte molecule^56^ and found that the presence of extended AuNP chains significantly increases the Raman signal, most likely due to plasmonic coupling between the neighboring AuNPs.^27,29^ Finally, by transferring the coating process to VA-SiNW arrays, we demonstrate the spatioselective assembly of AuNPs in different regions along the nanowires (Figure 1b) and explore the influence of geometric restrictions on the sensing performance of these substrates via SERS.

## Results and Discussion

The effect of different types of Si surface functionalization on the AuNP monolayer coverage and morphology was first investigated on flat Si substrates. Two types of Si surfaces were prepared: Si covered with amino-groups only, using APTES, and Si covered with both amino- and mercapto-groups, using a mixture of APTES and MPTMS. After functionalization, the Si substrates were incubated in an aqueous solution of citrate-stabilized AuNPs. Because functionalizing Si with MPTMS only led to a slow binding of the AuNPs under our experimental conditions (Figure S1), the use of pure MPTMS was not investigated further.

Aggregation of the AuNPs occurred on the APTES-functionalized Si, where the AuNPs formed large and ill-defined three-dimensional micron-sized aggregates that adsorbed at the Si surface (Figure S2). These are most likely driven by the release of residual silanes adsorbed at the Si surface after the functionalization step, which can bind to the Au surface and form siloxane shells and aggregates via condensation reactions.^61^ A 1 h washing step in deionized water prior to the self-assembly step solved this issue, which we found was less invasive than the use of ultrasound during the assembly step, as reported by other groups.^21,49^ This is particularly important for the self-assembly on SiNWs, because these tend to quickly break during ultrasonic treatment. Surfaces functionalized using an APTES/MPTMS mixture did not show the formation of such large micron-sized aggregates.^21^ However, large amounts of spherical structures form on the Si surface, with sizes of a few hundreds of nanometers, identified via energy-dispersive X-ray spectroscopy (EDX) as a byproduct of MPTMS (Figure S3). The density of these large C- and S-containing particles at the Si surface can be significantly reduced by placing the functionalized Si substrate on top of the AuNP solution, facing downward (Figure S4), as opposed to the standard incubation methods used by most groups, where the Si surface is placed at the bottom of the beaker, facing upward,^5,21,51^ or is dipped vertically into the AuNP solution.^4,23^

### AuNPs@APTES-Si Substrates

**Figure 2:**
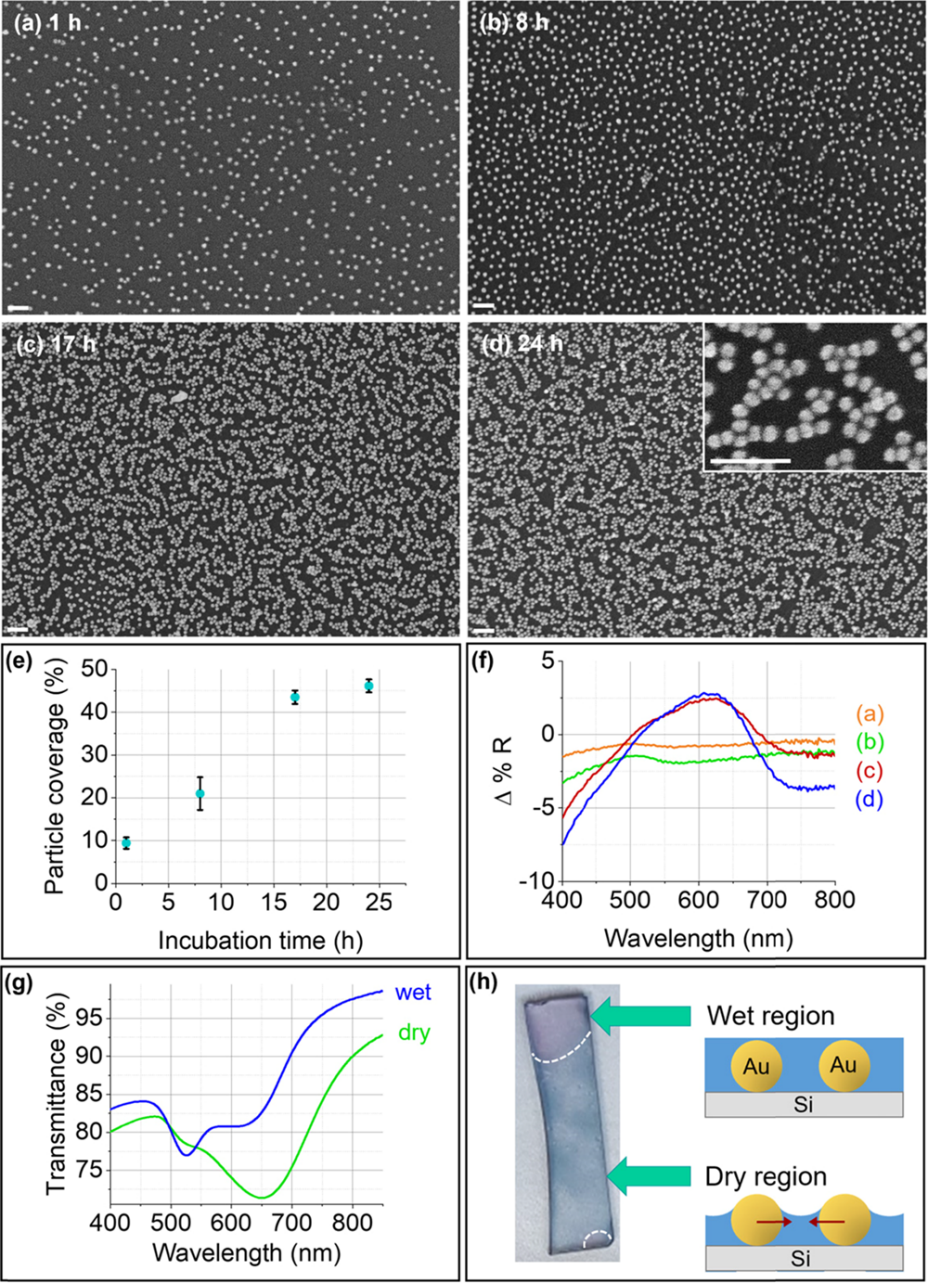
Flat Si functionalized with APTES after varying incubation times in the AuNP solution. (a–d) Secondary electron SEM images of the samples after (a) 1 h, (b) 8 h, (c) 17 h, and (d) 24 h. The scale bars correspond to 100 nm. (e) Particle coverage as a function of incubation time in the AuNP solution. (f) UV–Vis diffuse reflectance difference spectra of the AuNPs@APTES-Si samples shown in (a–d). (g and h) Investigation of the AuNP chain formation on an APTES-functionalized glass slide decorated with AuNPs (24 h incubation time): (g) Transmittance spectra in the wet (blue curve) and dried (green curve) state: (h) Photograph of the drying sample exhibiting wet and already dried regions, together with a scheme showing the proposed action of capillary forces on the AuNP movement during drying.

Figure 2a–d show typical secondary electron scanning electron microscopy (SEM) images of the AuNP monolayers formed on APTES-Si flat substrates. SEM analysis shows that the AuNP coverage increases for longer incubation times and plateaus after ca. 15 h (Figure 2e). The surface particle coverage is given as the relative area of the Si surface that is covered by AuNPs. At short incubation times (i.e., less than 8 h), a uniform monolayer of isolated AuNPs forms with AuNP coverages up to ca. 20%, leading to a continuous decrease in reflectance (Figure 2f). Because of the small AuNP size, the light extinction within isolated AuNPs is dominated by light absorption, with a negligible contribution of scattering.^25,62^ Thus, the decrease in reflectance observed for these samples around 550 nm is attributed to light absorption within the well-separated AuNPs, combined with the overall reduction of the reflective Si surface area due to the presence of the absorbing AuNPs. At longer incubation times, that is, 17 and 24 h, the AuNPs on the surface form interconnected AuNP chains which can be composed of up to ca. 100–200 AuNPs in close contact, with high AuNP coverages reaching ca. 42 and 45%, respectively. The diffuse reflectance spectra of these substrates show an increase in reflectance in the 500–700 nm range, which we attribute to the near-field coupling within the AuNP chains, known to have red-shifted LSPRs and an extinction dominated by scattering around the LSPR wavelength.^20,25,27^ The overall decreased reflectance below 500 nm can be attributed to increased absorption due to inter- and intraband transitions in Au and the decrease in the reflective Si surface area.^25,33^

To understand what causes the AuNP chain formation, a glass slide was functionalized with APTES and decorated with AuNPs (24 h incubation time). After rinsing with MilliQ water, the sample was immediately transferred into a cuvette filled with MilliQ water to keep it in the wet state. UV–Vis spectra were recorded in transmission mode of (i) the wet sample measured against MilliQ water containing a bare glass slide as reference, and (ii) the same sample after drying with an empty cuvette containing a dry bare glass slide in the reference beam. As can be seen in Figure 2g, the UV–Vis spectrum of the wet sample (blue curve) shows an intense extinction peak at ca. 525 nm, corresponding to the LSPR of isolated AuNPs, with a small shoulder above 600 nm, expected for interconnected AuNPs.^27^ In the dried-state sample (green curve), this trend is reversed: the UV–Vis spectrum is dominated by a broad peak at ca. 650 nm with a minor contribution at ca. 525 nm. These observations suggest the following: As long as the samples are wet, most of the AuNPs are far enough from each other to avoid a strong optical coupling; after drying, the isolated AuNPs are pushed together, forming interconnected AuNP chains that are responsible for the stronger extinction around ca. 650 nm. This aggregation process is most likely due to the capillary forces generated during the evaporation of the water film at the substrate surface.^63^ This is consistent with the absence of AuNP chains on silicon at low NP coverages (i.e., lower than ca. 20%, Figure 2), where the AuNPs are separated by large distances: The capillary forces are inversely proportional to the distance separating the two NPs and are thus too weak to induce AuNP chain formation in these samples.^63^ The AuNP chain formation can be followed by eye, where the originally purple wet sample becomes dark blue as the water film evaporates (shown in the photograph in Figure 2h).

### AuNPs@APTES/MPTMS-Si Substrates

**Figure 3:**
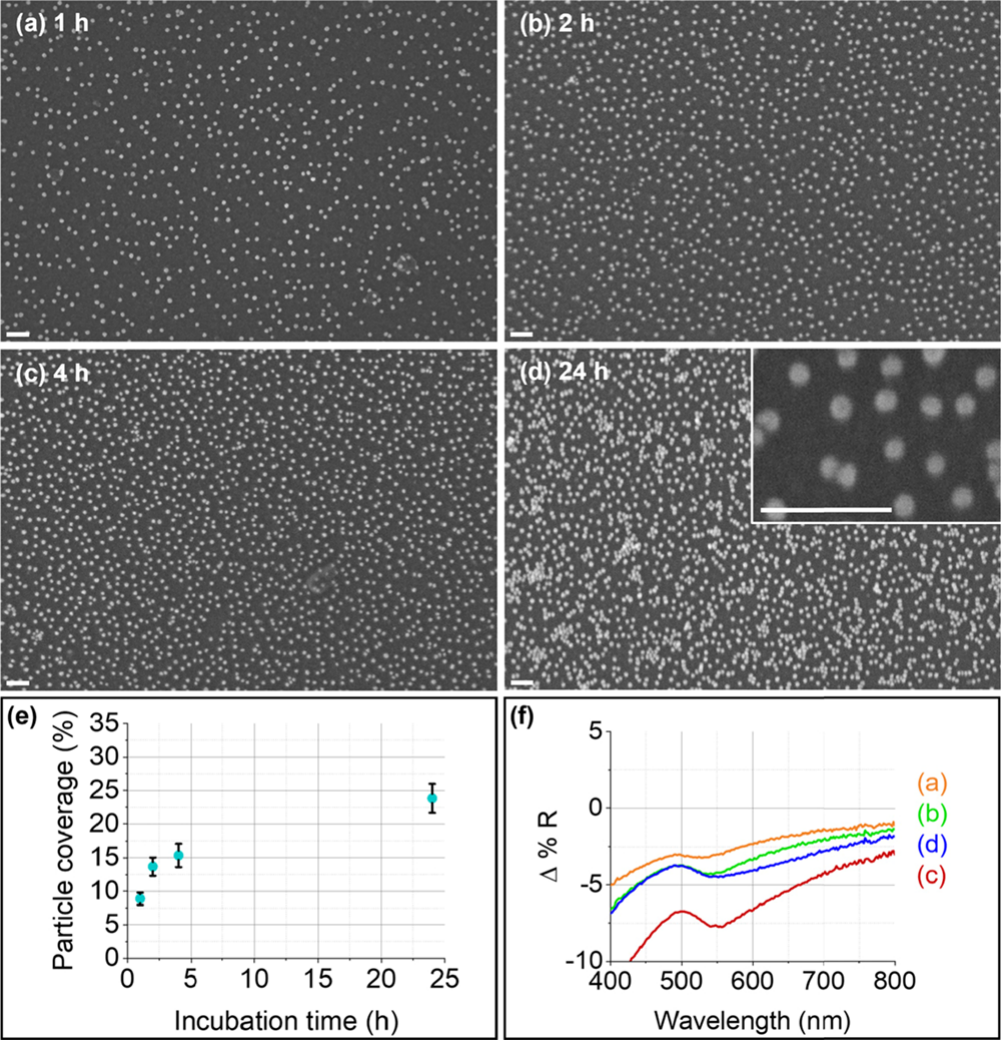
Flat Si functionalized with APTES/MPTMS after varying incubation times in the AuNP solution. (a–d) Secondary electron SEM images of the samples after (a) 1 h, (b) 2 h, (c) 4 h, and (d) 24 h. The scale bars correspond to 100 nm. (e) Particle coverage as a function of incubation time in the AuNP solution. (f) UV–Vis diffuse reflectance difference spectra of the AuNPs@APTES/MPTMS-Si samples shown in (a–d).

The AuNP monolayers forming on the APTES/MPTMS samples are composed almost exclusively of isolated AuNPs (Figure 3a–d) for all investigated incubation times (i.e., up to 24 h, corresponding to a NP coverage of ca. 25%, as shown in Figure 3e). These samples exhibit reduced reflectance for all incubation times (Figure 3f), attributed to the fact that the particles remained well-separated after self-assembly. Thus, no hotspots are formed, and absorption dominates over scattering due to the small size of the AuNPs.^25,27,62^ The dip in reflectance is observed at ca. 540 nm, similarly to the AuNPs@APTES-Si samples incubated for less than 8 h in the AuNP solution. The sample incubated for 24 h shows a slight degree of aggregation with the presence of a few AuNP clusters, which leads to a small increase in reflectance due to increased scattering by the AuNP aggregates compared to the sample incubated for 4 h.^27^

Compared to the mixed APTES/MPTMS-Si substrates, the ATPES-Si substrates showed a higher AuNP loading (coverage of ca. 25% vs 45% after 24 h, respectively) along with the formation of interconnected AuNP chains (compare Figure 3e with Figures 2e and 3d with Figure 2d). Taken together, these experiments show that the citrate-stabilized AuNPs bind faster to the charged amino-groups, attributed to the attractive electrostatic interaction between the positively charged protonated amino-groups of the APTES monolayer and the negatively charged deprotonated carboxylic acid-groups of the citrate.^21^ Under our experimental conditions, the covalent binding of the AuNPs to the mercapto-groups is slower, as shown by the inefficient assembly of the AuNPs after 17 h on a Si surface covered with mercapto-groups only (Figure S1).^21^ Although the reason for this slow binding is unclear, it could be due to the diffusion limited mass-transport of the AuNPs to the mercapto-groups. Thus, the MPTMS in the APTES/MPTMS surfaces most likely dilutes the density of rapidly binding positively charged amino-groups, which slows down binding, decreases the AuNP coverage, and limits the formation of interconnected AuNP chains seen at high coverages on the APTES substrates.^21^

### AuNPs@VA-SiNW Arrays

**Figure 4:**
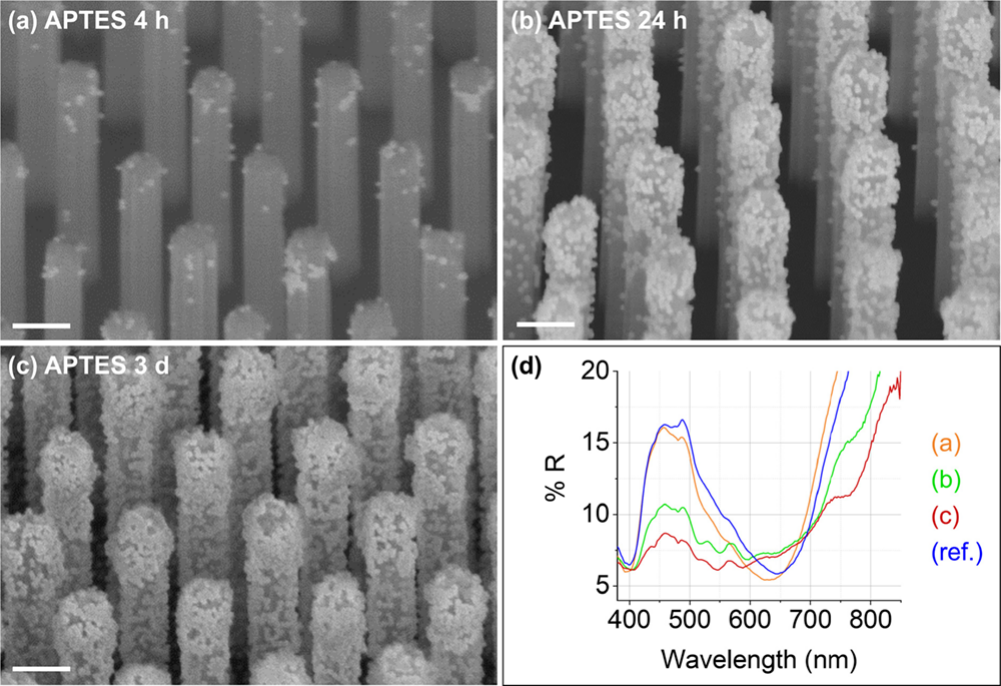
SiNWs functionalized with APTES after varying incubation times in a 1:1 mixture of AuNP solution and absolute ethanol. (a–c) Mixed secondary and back-scattered electron SEM images in tilted view (tilt angle = 45 °) of APTES-functionalized SiNWs after (a) 4 h, (b) 24 h, and (c) 3 days in a 1:1 mixture of AuNP solution and absolute ethanol. The scale bars correspond to 200 nm. (d) Corresponding UV–Vis diffuse reflectance spectra of the AuNPs@SiNWs shown in (a–c), along with the reference bare SiNW spectrum (blue curve, ref.).

Building on our findings on flat surfaces, we transferred the coating process to more complex geometries, namely, VA-SiNW arrays, to investigate the possibility to control AuNP assembly on Si surfaces not only in two dimensions but also in three dimensions. This not only increases the substrate surface area, but can also enhance the optical properties of the resulting composite to provide large and uniform E-field enhancements.^1,4,5,11,17,28^ VA-SiNW arrays, with an average pitch, nanowire diameter, and length of 407, 135, and 1340 nm, respectively, were prepared via colloidal lithography and metal-assisted chemical etching (MACE), as previously reported from our group (more details in the Experimental Section).^37,64^ Functionalization with APTES was carried out using the protocol developed for the fabrication of AuNPs@APTES-Si flat substrates. To enforce complete wetting of the nanostructured substrate with the colloidal dispersion, the AuNP self-assembly was carried out using a 1:1 mixture of AuNP solution and absolute ethanol. Figure 4a–c show representative secondary electron SEM images of the samples after incubation in the 1:1 mixture of AuNP solution and absolute ethanol for 4 h, 24 h, and 3 days, along with the corresponding diffuse reflectance spectra (Figure 4d).

**Figure 5:**
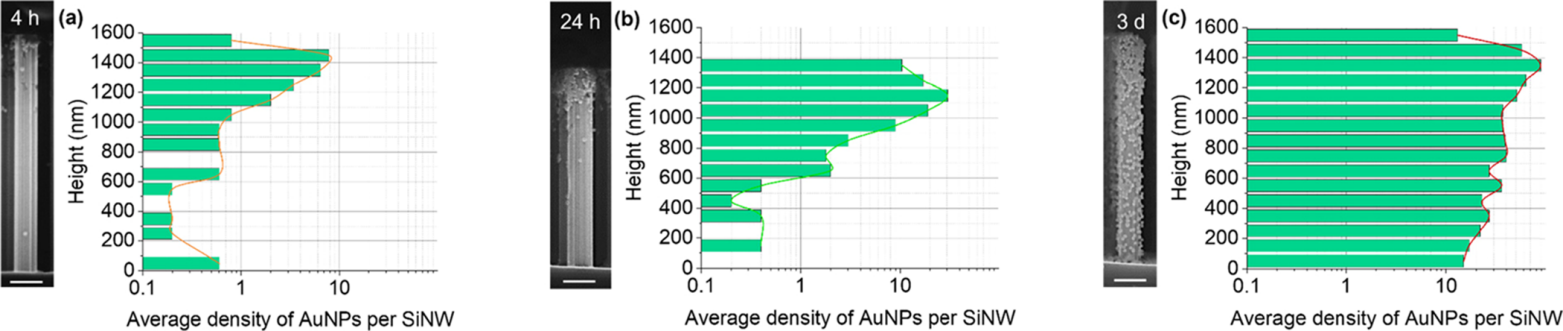
Spatial distribution of the AuNPs per SiNW along the nanowire long-axis for three different incubation times (log scale): (a) 4 h, (b) 24 h, and (c) 3 days, together with mixed secondary and back-scattered electron SEM images of one representative SiNW (scale bars: 200 nm).

After 4 h, the AuNPs are mostly isolated and located at the top of the SiNWs (Figure 4a), with the presence of small NP clusters composed of ca. 2–6 AuNPs in close contact. After 24 h, the AuNPs are distributed over the entire SiNW length, with some AuNPs also present at the Si flat bottom surface (Figure 4b). Most AuNPs are accumulated in larger AuNP clusters at the top of the nanowires, composed of up to ca. 50–60 AuNPs, which are reminiscent of the long AuNP chains that form on flat APTES-Si at high AuNP loadings. This demonstrates the preferential binding of the AuNPs to the top of the nanowires, attributed to the slow diffusion of the AuNPs into the nanowire array, which limits the amount of AuNPs and AuNP chains present in the subsurface nanowire region. Because AuNP chains form at high AuNP coverages during the drying process on flat Si and SiO_2_/glass surfaces (Figure 2), the AuNP chains and clusters observed on the SiNWs are also most likely forming due to the capillary forces generated during solvent evaporation. After a much longer incubation time (i.e., 3 days), the AuNPs densely coat the SiNWs: large AuNP clusters composed of up to ca. 250–300 AuNPs are observed at the top of the SiNWs, while small AuNP clusters, composed of ca. 2–10 AuNPs, are present at the bottom part of the SiNWs. The AuNP density is adjusted by the incubation time: After 4 h, 24 h, and 3 days of incubation, each SiNW is decorated with an average of ∼25, 95, and 590 AuNPs, respectively. Thus, our self-assembly conditions can be used to prepare both sparse and highly dense AuNP coatings on SiNWs, as shown in Figure 5 that presents a detailed evaluation of particle positions and densities along the nanowire long-axis. The higher NP density observed at the top of the nanowires is expected to drive the AuNP aggregation in this region. Additionally, SEM analysis shows that the SiNWs are covered with AuNP monolayers, with a very rare occurrence of multilayer formation after extended incubation times (see Figures S6 and S7 for additional SEM images). The SiNW array scaffold used here offers a periodic and well-defined topography that allows the self-assembly process to proceed uniformly, providing control over NP density and three-dimensional location (i.e., degree of NP clustering at the Si surface, and NP position along the SiNW long-axis) through the complete macroscopic substrate. Preparing such extended, periodic, and dense AuNP aggregate arrays is not easily done using conventional methods to nanostructure Au in three-dimensions, for example, dealloying,^65^ reduction of Au(I) networks,^66^ or direct NP-NP self-assembly,^67,68^ which highlights the versatility of our synthetic approach. To achieve reproducible AuNP density and position on the SiNWs, we recommend using a freshly opened silane bottle, which provides a reliable and efficient functionalization of the Si surface with amino-groups. Indeed, the use of aged silanes, stored in the laboratory for more than 1 year, led to lower AuNP densities (cf. Figure S5).

Figure 4d shows the corresponding diffuse reflectance spectra of the AuNPs@SiNWs samples. VA-SiNWs are known to sustain so-called leaky waveguide modes leading to an increased absorption at specific wavelengths, which can be seen by well-defined dips in the UV–Vis diffuse reflectance spectra.^8,22^ Two leaky waveguide modes can be observed at around 400 and 650 nm for all the nanowire arrays (Figure 4d).^8,22^ This demonstrates the high degree of homogeneity of the produced VA-SiNW arrays. The addition of the AuNPs leads to an overall decrease in reflectance across the 400–800 nm range compared to the bare VA-SiNW arrays. This effect is more pronounced with higher densities of AuNPs. Similarly to the flat Si substrates, the decrease in reflectance is attributed to absorption in the AuNPs and the reduction of the reflective Si surface area.^25,62^ Compared to the 4 h sample, the samples incubated for 24 h and 3 days show a slight increase in reflectance approximately between 600 and 700 nm. This observation is consistent with the results obtained on the AuNPs@APTES-Si substrates formed with long incubation times (≥17 h), where the presence of long AuNP chains composed of AuNPs in close contact caused an increase in reflectance and a red-shift of the coupled AuNP LSPR.^20,25,27^

### Raman Measurements

**Figure 6:**
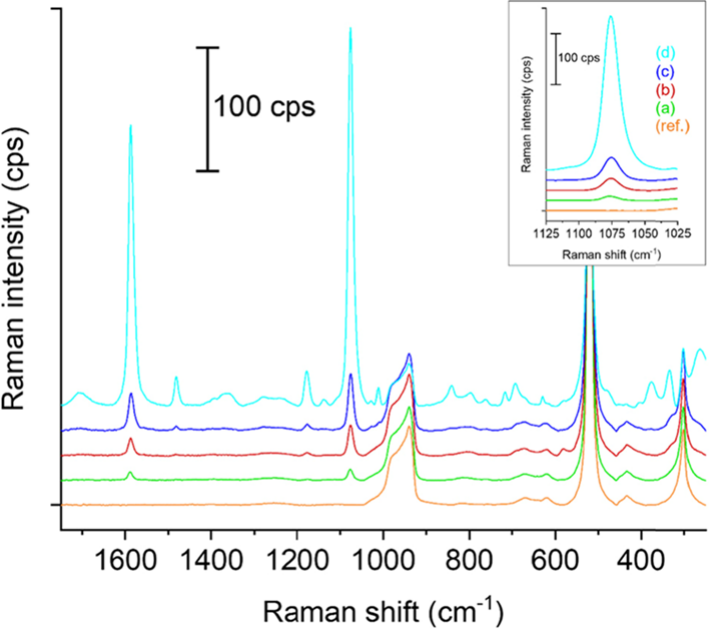
Baseline-corrected and offset Raman spectra of 4-MBA on flat Si substrates functionalized with either APTES or APTES/MPTMS and incubated in AuNP solution for 4 and 24 h, along with the spectrum of a reference sample (APTES-Si without AuNPs, orange curve, (ref.)). The spectra have been obtained at a laser excitation wavelength of 785 nm and a laser power of 10 mW on the sample, and the mean of 25 measurements on 25 different positions is shown for each sample. (a) APTES-functionalized Si incubated in AuNP solution for 4 h. (b) APTES/MPTMS-functionalized Si incubated in AuNP solution for 4 h. (c) APTES/MPTMS-functionalized Si incubated in AuNP solution for 24 h. (d) APTES-functionalized Si incubated in AuNP solution for 24 h. The inset shows a magnification of the Raman peak belonging to the analyte molecule 4-MBA, positioned at a Raman shift of ca. 1075 cm^–1^.^56^

The different AuNPs@Si substrates were investigated via confocal Raman microscopy. Figure 6 shows the Raman results for the AuNPs@APTES-Si and the AuNPs@APTES/MPTMS-Si flat substrates incubated in the AuNP solution for 4 and 24 h, each, along with a reference spectrum of a flat Si substrate functionalized only with APTES molecules (APTES-Si; Figure 6, orange curve). The Raman spectra were recorded at a laser excitation wavelength of 785 nm and a laser power of 10 mW on the sample, after deposition of a droplet of 1 mM 4-MBA as the analyte molecule onto the substrate surface (details in the Experimental Section). The strongest peak of the Raman spectrum of 4-MBA at ca. 1075 cm^–1^, which originates from a vibrational mode of the aromatic ring, was chosen to compare the SERS activity of the different substrates.^56^ For both types of silane monolayers (i.e., APTES and mixed APTES/MPTMS), the samples incubated for 4 h in the AuNP solution provided the lowest Raman intensities (Figure 6, green and red curves; Table S1). The higher Raman signal measured on the AuNPs@APTES/MPTMS-Si substrate can be explained by the higher AuNP coverage observed after 4 h incubation, measured to be 7.9% for the AuNPs@APTES-Si and 15.4% for the AuNPs@APTES/MPTMS-Si substrates used for the Raman investigation. Similarly, the slightly higher NP coverage of the AuNPs@APTES/MPTMS-Si substrate incubated for 24 h, that is, around 25%, accounts for the slightly higher Raman signal measured on this sample. However, after 24 h, the AuNPs@APTES-Si substrate provides a Raman signal that is more than 30 times higher compared to the same sample incubated only for 4 h (see Table S1), even though the AuNP coverage is only ca. six times higher. Additionally, it also has the lowest relative standard deviation (std) in the Raman signal of all the samples measured, i.e., 22%, to be compared with relative stds in the range of 40–99% for the other AuNPs@Si flat substrates. A summary of the actual peak intensities and the relative stds for all investigated substrates is provided in Table S1. As suggested by the secondary electron SEM images and the UV–Vis measurements, the AuNP chains formed on this sample (AuNPs@APTES-Si incubated for 24 h) are likely to provide a large density of hot spots in between the AuNPs in close contact, which we believe is key to provide large and reliable Raman signal enhancements.^25,27^ This is supported by our finite-difference time-domain (FDTD) simulations. Thus, the controlled assembly of the AuNPs into interconnected chains provides a uniform density of hot spots leading to a higher SERS activity and more reproducible measurements.

**Figure 7:**
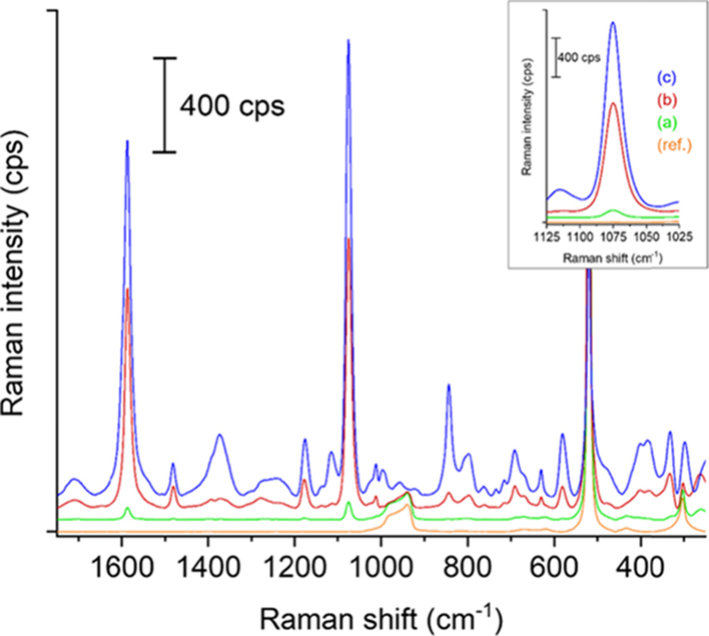
Baseline-corrected and offset Raman spectra of 4-MBA on VA-SiNW array substrates functionalized with APTES and incubated in a 1:1 mixture of AuNP solution and absolute ethanol for (a) 4 h, (b) 24 h, and (c) 3 days, along with the spectrum of a reference sample (SiNWs without AuNPs, orange curve, (ref.)). The spectra have been obtained at a laser excitation wavelength of 785 nm and a laser power of 10 mW on the sample, and the mean of 25 measurements on 25 different positions is shown for each sample. The inset shows a magnification of the Raman peak belonging to the analyte molecule 4-MBA, positioned at a Raman shift of ca. 1075 cm^–1^.^56^

Raman spectra were also recorded on the various AuNP-decorated VA-SiNW arrays (Figure 7). The reference bare VA-SiNW sample (APTES-functionalized SiNWs after the washing step, no AuNPs; orange curve) does not show any signal from the analyte molecule 4-MBA. As expected from previous reports, the AuNPs@SiNWs samples show a large Raman signal from the analyte molecule 4-MBA.^4,5,11,56^ The signal intensity measured on the AuNPs@SiNWs incubated for 24 h and 3 days in the 1:1 mixture of AuNP solution and absolute ethanol is much higher than on the AuNPs@SiNWs incubated for 4 h, most likely due to the higher amount of isolated AuNPs and AuNP chains that are present after longer incubation times. The AuNPs@SiNWs sample incubated for 3 days shows the highest signal enhancement, which is around twice the signal measured on the AuNPs@SiNWs incubated for 24 h and 25 times that on the AuNPs@SiNWs incubated for 4 h, respectively.

**Figure 8:**
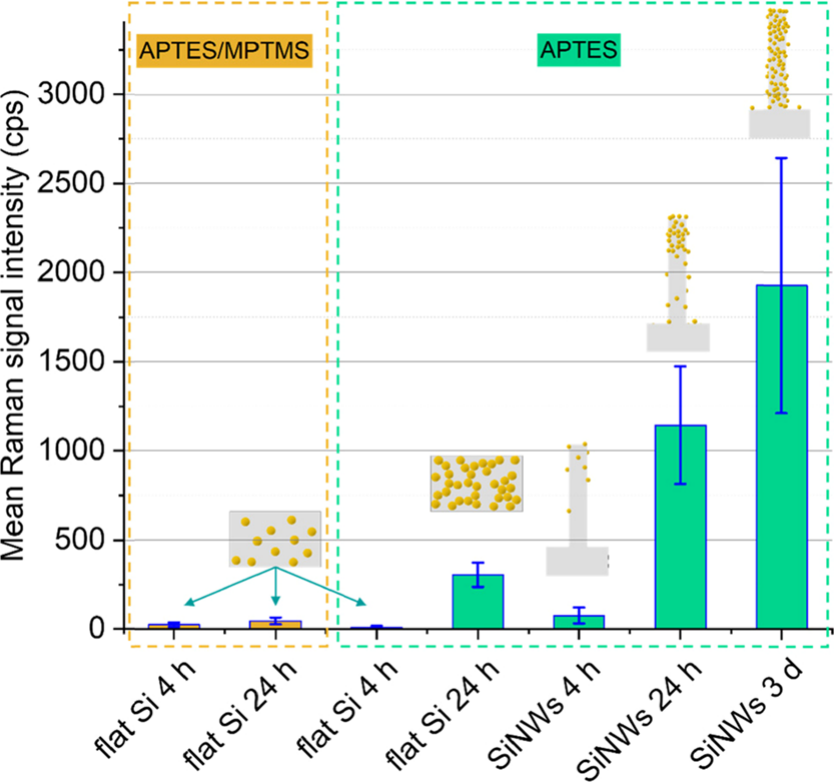
Mean signal intensity of the Raman peak belonging to the analyte molecule 4-MBA positioned at a Raman shift of ca. 1075 cm^–1^, for different flat Si and VA-SiNW substrates decorated with AuNPs as indicated in the figure.^56^ The spectra have been obtained at a laser excitation wavelength of 785 nm and a laser power of 10 mW on the sample, and the mean of 25 measurements on 25 different positions is shown for each sample. The respective standard deviation is indicated by the blue bars.

Figure 8 compares the intensity and std of the Raman signal for the analyte molecule 4-MBA measured at ca. 1075 cm^–1^ on the different flat Si and VA-SiNW arrays decorated with AuNPs. AuNPs@SiNWs samples are more sensitive SERS substrates at comparable numbers of AuNPs per apparent surface area exposed to the laser beam, assuming a flat topography for all samples. For instance, the Raman signal measured on the AuNPs@SiNWs (24 h, ∼650 AuNPs/μm^2^, with AuNP chains) is almost 50 times higher than the one measured on AuNPs@APTES/MPTMS-Si (flat, 4 h, ∼680 AuNPs/μm^2^, isolated NPs), although the NP density is almost identical, and four times higher than the Raman signal measured on the AuNPs@APTES-Si (flat, 24 h, ∼1980 AuNPs/μm^2^, with AuNP chains), which has a ca. three times higher NP density than the nanostructured SiNW sample. This shows that SiNW arrays can maximize the E-field enhancement on self-assembled AuNP monolayers, therefore providing higher Raman signal intensities.

Overall, the SERS activity of the different samples investigated ranks in the following order: AuNPs@APTES-Si (4 h) < AuNPs@APTES/MPTMS-Si (4 h) < AuNPs@APTES/MPTMS-Si (24 h) < AuNPs@SiNWs (4 h) < AuNPs@APTES-Si (24 h, AuNP chains) < AuNPs@SiNWs (24 h) < AuNPs@SiNWs (3 days). Interestingly, the AuNPs@APTES-Si flat substrate incubated for 24 h, which is covered with a monolayer of interconnected AuNP chains, provides a higher SERS activity than the AuNPs@SiNWs substrate incubated for 4 h (Figure 8). This demonstrates an approach that is perhaps simpler, although not as effective, to achieve a large enhancement of the Raman signal by forming AuNP chains on flat substrates, without requiring VA-SiNW arrays.

### Electromagnetic Simulations

**Figure 9:**
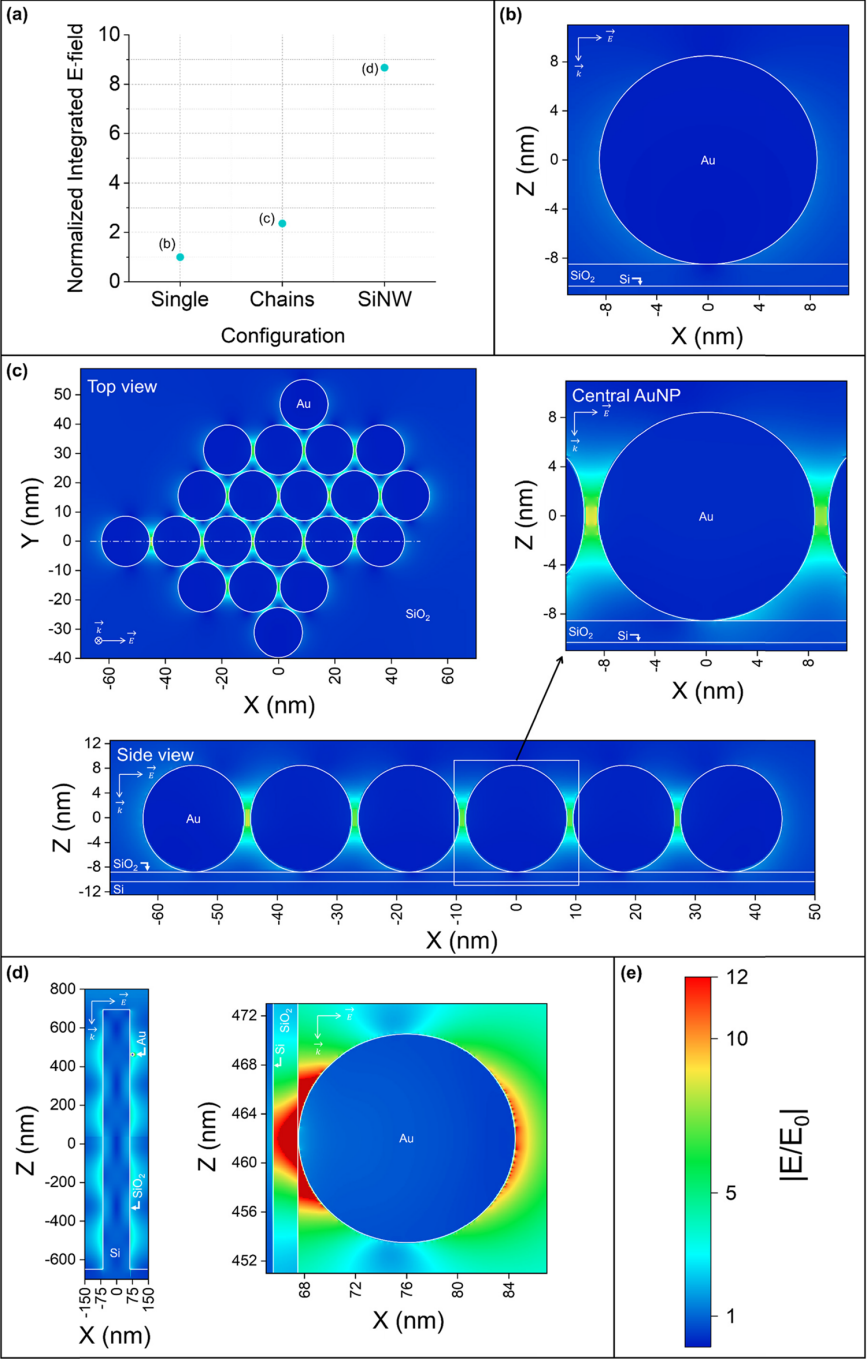
Electromagnetic FDTD simulations. (a) Integrated surface E-field strength around a single AuNP on flat Si, a central AuNP in a group of 20 AuNPs on flat Si, and a single AuNP on a SiNW located in a region of increased E-field intensity, normalized to the value obtained for the single AuNP on flat Si. (b) E-field map of a single AuNP on flat Si. (c) E-field maps of an aggregate of 20 AuNPs on flat Si in top and side views and magnified E-field map of a central AuNP as indicated in the figure. (d) E-field maps (overview and detail) of a single AuNP on a SiNW in a region of increased E-field intensity. (e) Color-coded E-field enhancement scale of the E-field maps shown in (b–d).

To further elucidate the origin of these differences, we performed three-dimensional electromagnetic FDTD simulations at the Raman excitation wavelength (i.e., 785 nm, simulation details in the Experimental Section) on three different Au/Si architectures: an isolated AuNP on Si, interconnected AuNPs on Si, and a AuNP on a SiNW. Figure 9a compares the integrated E-field strength over a 1 nm thick shell around the AuNP surface for the three configurations. It is significantly higher around the AuNP located on the SiNW, which explains the higher Raman signals measured on nanostructured Si, scaling as |*E*/*E*_0_|^4^, where *E*_0_ and *E* are the incident and surface E-field amplitudes, respectively.^33^ The relatively low Raman signal measured on the AuNPs@SiNWs (4 h) might be explained by its low AuNP coverage (i.e., ∼170 AuNPs/μm^2^—apparent area), which decreases the probability for efficiently binding the 4-MBA during the few seconds required for the analyte droplet to dry at the sample surface. The E-field maps (Figure 9b–e) show that the E-field is enhanced most at the AuNP/SiNW interface for the Raman excitation wavelength used in this work. Because these simulations were performed with one E-field polarization only, they are not an accurate representation of the actual Raman experiment. Additionally, the E-field enhancement of the AuNP/SiNW substrate was simulated by locating the AuNP in the local maximum E-field generated by the SiNW. Because the E-field along the SiNW is periodic due to Fabry-Pérot-like resonances,^22,69^ most AuNPs coating the SiNW experience a lower E-field intensity. However, the clear trend observed with these simulations is in line with the relative changes in the Raman signal measured on these different Au/Si substrates.

## Conclusions

To conclude, a solid synthetic protocol to reliably functionalize flat and nanostructured Si surfaces with different silane monolayers (APTES and APTES/MPTMS), used for the deposition of AuNP monolayers, is described. The influence of various synthetic parameters on the AuNP monolayer morphology, density, and uniformity is provided, along with the resulting optical properties and sensing performance via SERS. On flat Si, mixed APTES/MPTMS layers consistently yield isolated AuNPs, while pure APTES-functionalized Si leads to higher AuNP coverage and provides the opportunity to reproducibly prepare monolayers of interconnected AuNP chains. Our results suggest that the formation of these AuNP chains is driven by capillary forces during the drying process. The effect of AuNP coverage and aggregation state on the reflectance behavior of the AuNPs@Si surface is provided, while Raman measurements show increasing signal strengths for increasing amounts of AuNPs, as well as a strong SERS effect of the interconnected AuNP chains due to near-field coupling^25,27^ that also provides a more reproducible Raman signal intensity. Additionally, the spatioselective deposition of AuNPs in specific regions of the SiNWs is demonstrated along with a control over the AuNP density and monolayer morphology (i.e., isolated vs nearly touching AuNPs). The resulting optical properties and SERS activities of these substrates are reported, which confirms the higher SERS activity of the AuNPs@VA-SiNW arrays compared to the flat substrates at similar AuNP coverages. Our FDTD simulations show a large E-field enhancement at the AuNP/SiNW interface at 785 nm, which explains the large Raman signal measured on the AuNPs@VA-SiNW array substrates. Thus, our work presents two different synthetic pathways to obtain highly sensitive SERS sensors by either (i) constraining the two-dimensional assembly of AuNPs into chains on a flat Si surface or (ii) assembling the AuNPs in three-dimensions on VA-SiNW arrays. Although the two-dimensional assembly of AuNPs into chains provides a considerable enhancement of the Raman signal, the highest enhancements were achieved by assembling AuNPs into dense coatings around and along VA-SiNWs.

## Experimental Section

### Materials

All chemicals listed here were used without further processing, unless stated otherwise. APTES, MPTMS, 4-MBA, HAuCl_4_·3H_2_O, iodine, potassium iodine, and anhydrous toluene (99.8%) were purchased from Sigma-Aldrich. Acetone (99%), calcium chloride, ethanol (96%), isopropanol (IPA) (≥98%), absolute ethanol (99.96%), and hydrofluoric acid (40%) were acquired from VWR. Hydrogen peroxide (30%) and tri-sodium citrate dihydrate were obtained from Merck. Sulfuric acid (95–97%) was supplied by Supelco. The MilliQ water used was double deionized using a MilliQ system with a resistivity of 18 MΩ. N-doped Si wafers (<100>, resistivity 1–30 Ω cm) were obtained from Si Materials, Germany. For the synthesis of SiO_2_@PNiPAm core–shell particles used as a colloidal template, *N,N*′-methylenebis(acrylamide) (99%), ammonium persulfate (98%), ethanol (99.9%), hexane (≥99%), tetraethyl orthosilicate (98%), ammonium hydroxide solution (28–30% NH_3_ basis), (3(trimethoxysilyl)propyl methacrylate) (98%), fluorescein isothiocyanate isomer I (>90%), and (3-aminopropyl)triethoxysilane (>98%) were purchased from Sigma-Aldrich and used as received. *N*-Isopropylacrylamide (97%) was obtained from Sigma-Aldrich as well but purified before usage by recrystallization from hexane (95%, Sigma-Aldrich).^70^

### Preparation of SiO_2_@PNiPAm Core–Shell Particles

SiO_2_ NPs were prepared according to the Stöber process in a procedure which is described in detail in the literature.^70,71^ 5.8 mg of fluorescein isothiocyanate isomer I were dissolved in 1.5 mL of EtOH corresponding to a 10 mM solution. Then, 26.19 μL of APTES (10 equiv) were added, and the mixture was stirred in the dark for 2.5 h. 900 μL of this mixture were diluted in 4.50 mL EtOH (5 vol equiv) yielding the final fluorescent dye solution used for labeling of the SiO_2_ NPs. Functionalization of the SiO_2_ NPs with (3(trimethoxysilyl)propyl methacrylate) was carried out according to the literature.^70^ Surfactant-free precipitation polymerization in a nitrogen atmosphere was used for the polymerization of the PNiPAm-microgel shell on the functionalized SiO_2_ NPs. 282.9 mg of *N*-isopropylacrylamide were dissolved in 45 mL MilliQ water in a round-bottom flask to produce a 50 mM solution together with 9.636 mg *N*,*N*′-methylenebis(acrylamide). The core–shell particles were purified from free microgel particles and pure SiO_2_ NPs by centrifugation with MilliQ water.^70^

### Preparation of the SiNWs

SiNW arrays were synthesized using a combination of colloidal lithography^70,72^ and MACE,^37,64^ as previously done in our group. Shortly, core–shell SiO_2_–PNiPAm nanoparticles, with an average diameter of the SiO_2_ core of 160 nm, and of the polymeric shell of 369 nm (hydrodynamic diameter at 50 °C) were assembled on Si (precleaned by ultrasonication in acetone and IPA and a 7 min oxygen plasma etching (OPE) treatment (100 W, 4 sscm, Femto SLS, Diener, Germany) to ensure hydrophilicity) via Langmuir–Blodgett trough.^63,70^ In this step, a mixture containing clean EtOH and the core–shell NP suspension in a 1:1 ratio was used. The assembly was done at 90° to the air-water interface. The polymeric shell was then completely etched via oxygen plasma during 12 min at 50 W. An adhesion layer of aluminum-doped zinc oxide was deposited in a Clustex 100 M sputtering system by Leybold Optics for 1 s at 75 W followed by another sputtering step. Here, Au was sputtered for 200 s at 40 mA using the Cressington Sputter Coater 108 auto. The SiO_2_ spheres were teared off using adhesive tape leaving behind a Au nanohole film. The samples were etched for 8 min in a MACE solution containing 10 mL H_2_O, 10 mL HF and 0.75 mL H_2_O_2_ and rinsed three times in MilliQ water. The samples were then etched in a 20 mL H_2_O and 4 mL HF mixture to remove any residual porous SiO_2_ on the SiNW surface. After rinsing the samples three times in MilliQ water and once in ethanol, the samples were dried in air.

### Synthesis of AuNPs

AuNPs were synthesized following the citrate reduction process:^57−60^ 25 mg of dry HAuCl_4_·3H_2_O were dissolved in 250 mL MilliQ water to obtain the Au stock solution. 90 mL of the Au stock solution were transferred into a 250 mL two-neck round-bottom flask together with a stir bar and heated to boil under reflux in an oil bath. 5 mL of 1 wt% citrate solution were added with a pipette. After 5 min, the stirring was stopped, whereas boiling was continued for another 25 min to complete the reduction of all gold ions. A color change from transparent to wine red indicates the formation of AuNPs in solution. The size of the AuNPs was determined to be around 17 nm, and the measured UV–Vis transmittance spectrum of the AuNP solution is given in Figure S8.

### Silane Functionalization

The Si wafer was cut into pieces of roughly 4 × 2.5 cm^2^, which were sonicated in acetone and IPA for 5 min each, followed by an OPE step at 50 W for 10 min. In case of the SiNWs, the first step included a dissolution of the Au film at the bottom using KI/I_2_ solution (10 g KI and 5 g I_2_ dissolved in 85 g MilliQ water) for 80 min, followed by three washing steps, twice in MilliQ water and once in ethanol before they were also treated via OPE at 50 W for 10 min. The next steps were identical for flat Si and SiNWs. Here, the samples were treated in piranha solution (*Caution: 3:1 mixture of sulfuric acid H_2_SO_4_ and 30% hydrogen peroxide H_2_O_2_; piranha solution is extremely corrosive and strongly oxidizing for organics; explosive if not handled with care*),^73^ composed of 30 mL H_2_SO_4_ and 10 mL H_2_O_2_ for 30 min and subsequently rinsed with MilliQ water thoroughly. In the following, an OPE treatment at 100 W for 30 min finished the hydroxylation of the surfaces. At this point, the samples could be stored in MilliQ water.^53^

For the reaction with the silane, the samples were dried under ambient conditions and immersed in a round bottom flask containing 50 mL anhydrous toluene and the silane(s) in the desired concentration. For a 50 mM solution, 584 μL of APTES or 292 μL of APTES concomitantly with 232 μL MPTMS, respectively, were added to 50 mL of anhydrous toluene. Subsequently, the system was heated up in an oil bath and under reflux to 90 °C. As soon as a temperature of 90 °C was reached, the temperature was held constant for the next 5 h and afterward switched off. The round bottom flask containing the samples was removed from the oil bath and allowed to cool down still under reflux. On the following day, the samples were removed from the round bottom flask and sonicated in 20 mL of fresh anhydrous toluene for 10 min. After sonication, the samples were again transferred into 20 mL fresh anhydrous toluene and kept for 1 h. The SiNWs were directly transferred into 20 mL fresh anhydrous toluene for 1 h and not sonicated before. Here, we found out that there was no difference if the samples have been washed in toluene for 3 days or 1 h. Next, the samples were annealed in an already preheated oven for 2 h at 90 °C. To verify that the functionalization of the surfaces was successful, contact angle measurements with water were carried out on a KRÜSS DSA10 contact angle measurement device. The results were evaluated by the software Drop Shape Analysis_1_91. As expected, the cleaned and hydroxylated Si surfaces were highly hydrophilic, whereas the functionalized Si surfaces were significantly more hydrophobic, as evidenced by an increased contact angle of the water droplet.^21^

For the functionalization of VA-SiNWs and the subsequent investigation of their optical properties, APTES was the silane of choice because the formation of the spherical silane byproducts produced by the MPTMS and their possible undesired influence on the measured optical properties should be avoided.

Nb: The AuNPs were found to bind faster to the functionalized surface when we used fresh APTES and MPTMS (i.e., from a freshly opened bottle), as opposed to using silanes from bottles that were already opened.

### AuNP Self-Assembly

Finally, the samples were cut into smaller pieces of ca. 1 × 1 cm^2^ and immersed in AuNP solution for varying times. SiNW samples were cut in even smaller pieces and immersed in a 1:1 mixture of AuNP solution and absolute ethanol. APTES-functionalized samples were immersed into MilliQ water for 1 h before incubation in AuNP solution. In case of SiNWs, the washing procedure consisted of rinsing with ethanol followed by 1 h incubation in MilliQ water, and both of those steps were repeated a second time. The incubation in AuNP solution was started immediately after the washing procedure as soon as the samples had dried in air. The incubation time of the SiNWs in AuNP solution was varied between 4 h, 24 h, and 3 days (i.e., 72 h).

### Diffuse Reflectance UV–Vis Spectra

UV–Vis spectra were recorded using a PerkinElmer Lambda 1050. The diffuse reflectance measurements were performed using a 150 mm integration sphere and a white Spectralon reference for the baseline correction. During sample measurement, a black reference was placed behind the sample to avoid the influence of light reflection at the back of the samples. For samples which were too small to be measured in the usual setup of the integration sphere, a circular pinhole of 5 mm (flat Si samples) or even 3 mm (SiNW samples) in diameter together with a light focusing lens and a circular aperture to adjust the size of the light beam to the smaller sample area was used. The correct alignment of the light beam on the sample was manually adjusted before each measurement series by adapting the size of the circular aperture for the correct size of the beam and turning the reflecting mirror for positioning the light beam on the sample. The alignment was properly done if the light beam hits exactly the pinhole and is centered in it. The measurement range was set between 300 and 850 nm using a step width of 2 nm.

Regarding the UV–Vis measurements depicted in Figures 2f and 3f, the UV–Vis spectrum of a reference sample was subtracted from each of the substrates measured (i.e., an APTES-functionalized Si surface incubated in MilliQ water for 1 h was subtracted from the AuNPs assembled on APTES-functionalized samples, and by analogy an APTES/MPTMS-functionalized Si surface from the AuNPs assembled on APTES/MPTMS-functionalized samples). Here, the difference spectra are shown; positive peaks correspond to increased reflectance compared to the reference samples, whereas negative peaks can be attributed to decreased reflectance. The original spectra can be found in Figure S9.

### SEM Images

SEM images were acquired using a Zeiss Ultra Plus 55 equipped with a field emission gun and Gemini lenses. The InLens SE detector was used to obtain topographical images, and the angle-selective back-scattered electron (AsB) detector was used to detect back-scattered electrons and obtain compositional contrast. The SEM images of AuNPs@SiNWs shown in this work were obtained by mixing the signals of both detectors. EDX measurements were acquired using a 50 mm^2^ silicon drift EDX detector from Oxford instruments.

The SEM images were analyzed with the freely available software ImageJ. The AuNP coverage was obtained by measuring the AuNP total area on the image, based on the difference in contrast between the bright AuNPs and the dark silicon surface. This number was then divided by the total image area. For every sample, two images were evaluated twice, each, in order to reduce the influence of experimental error induced by manually setting the image contrast. To estimate the height distribution of AuNPs on the SiNWs, the distance of single particles to the bottom of the SiNW array (i.e., the bulk Si) was measured on a known number of SiNWs on two different cross-sectional SEM images representative for the respective sample. The number of AuNPs visible in the SEM cross-sectional images within 200 nm long sections was counted. This number was then multiplied by two to consider the fact that only one side of the SiNWs can be viewed on one SEM image. The average AuNP density was then obtained by dividing the total number of AuNP counted by the number of SiNWs investigated. AuNPs sitting on the top of the SiNWs as well as those on the bottom flat Si were not counted.

### Raman Measurements

Raman spectra were recorded using a dispersive Thermo DXR2 Raman microscope (Thermo, USA) equipped with the confocal microscope BX41 by Olympus Corp. (Japan). The samples were first briefly cleaned in oxygen plasma (1 min at 20 W). A 2 μL droplet of the 1 mM solution of 4-MBA in IPA was then drop-cast and left to dry before the Raman measurement. The drop-cast approach was chosen instead of a complete functionalization in the 4-MBA solution because we found out that incubation in the analyte solution for an extended period of time (i.e., 18 h) can lead to a change in the structure of the AuNP monolayers, where a significant amount of AuNPs moves and accumulates into large AuNP aggregates. The Raman signal measured in such a case is not representative of the original Au/Si structure synthesized and studied in this manuscript. A laser excitation wavelength of 785 nm with a power of 10 mW was used, together with a 10× objective producing a laser spot with 3.1 μm diameter. A confocal microscope setup was used with a 50 μm pinhole entrance slit to the spectrometer, leading to a spectral resolution between 4.7 and 8.7 cm^–1^ over the whole spectral range recorded from 200 cm^–1^ to 3300 cm^–1^. The exposure time was set to 5 s with 9 accumulations of every spectrum. Twenty-five spectra per sample were acquired at different locations on the sample surface. The mean of the 25 baseline-corrected spectra is shown. Based on the mean of those 25 baseline-corrected spectra the relative stds in signal intensity were calculated for the Raman peak belonging to the analyte molecule 4-MBA, positioned at a Raman shift of ca. 1075 cm^–1^.^56^ Before recording every Raman spectrum, the autofocus function of the Raman instrument was used, which optimizes the excitation laser focal point to obtain the largest Raman signal.

### Electromagnetic FDTD Simulations

Electromagnetic FDTD simulations were performed using the software from Lumerical Solutions Inc., Vancouver, Canada. The dielectric functions of the utilized materials were used from the Lumerical materials library that is contained in the software. During the simulations a distance between the simulated structures and the simulation boundaries of at least λ/2 was used. Boundary conditions were set to periodic along *x*- and *y*-axes in case of infinitely extended VA-SiNW arrays. Perfectly matched layers were used otherwise. The refractive index of the surrounding medium was adjusted to 1. On top of the modeled Si structures, a 2 nm thin SiO_2_ layer was added to mimic the naturally grown oxide layer on top of Si surfaces. The simulated structures were illuminated with linearly polarized light by a plane wave source in a wavelength range from 400 to 850 nm. The mesh size was set to 0.1 nm (2D mesh) around the AuNPs to obtain high-resolution E-field maps. During simulation of the integrated E-field, a 0.2 nm mesh size (3D mesh) was used around the AuNPs in order to reduce the memory requirements of the simulation. The script of the advanced power analysis group provided by Lumerical was modified to extract and integrate the E-field components over the volume of the analysis group. With this, the contribution of the 1 nm thin shell around the AuNP, which is a measure for the overall surface E-field relevant for SERS, could be extracted.^74^

## Supporting Information Available

Additional SEM images, EDX data, UV–Vis transmittance spectrum of the AuNP solution and the original UV–Vis spectra of the AuNPs@Si flat substrates, and a table summarizing the signal intensities and relative stds in Raman signals (PDF)

## Abbreviations

2D: two-dimensional or two-dimensions
3D: three-dimensional or three-dimensions
4-MBA: 4-mercaptobenzoic acid
APTES: (3-aminopropyl)triethoxysilane
AsB: angle-selective back-scattered electron
Au: gold
AuNP: gold nanoparticle
BSE: back-scattered electron
CA: contact angle
EDX: energy-dispersive X-ray spectroscopy
E-field: electric field
FDTD: finite-difference time-domain
IPA: isopropanol
LSPR: localized surface plasmon resonance
MACE: metal-assisted chemical etching
MPTMS: (3-mercaptopropyl)trimethoxysilane
NP: nanoparticle
OPE: oxygen plasma etching
PNiPAm: poly(*N*-isopropylacrylamide)
SE: secondary electron
SERS: surface-enhanced Raman spectroscopy
SEM: scanning electron microscopy
Si: silicon
SiNW: silicon nanowire
std.: standard deviation
UV–Vis: ultraviolet–visible
VA-SiNW: vertically aligned silicon nanowire
