# Supplementary material for: Self-Assembled Au Nanoparticle Monolayers on Silicon in Two- and Three-Dimensions for Surface-Enhanced Raman Scattering Sensing
Source: ACS Appl Nano Mater. 2022 Aug 15;5(8):11839–51. doi: 10.1021/acsanm.2c01904 (PMC9425434; doi:10.1021/acsanm.2c01904)
Supplement: Supplementary file 1 — an2c01904_si_001.pdf [file an2c01904_si_001.pdf]

Supporting Information

Self-Assembled Au Nanoparticle Monolayers on Silicon in Two- and Three-Dimensions for Surface-Enhanced Raman Scattering Sensing

Theresa Bartschmid, [1] *Amin Farhadi,* [1] *Maurizio E. Musso,* [1] *Eric Sidney Aaron Goerlitzer,* [2] *Nicolas Vogel,* [2] *Gilles R. Bourret* [1,*]

[1] Department of Chemistry and Physics of Materials, University of Salzburg, Jakob Haringer Strasse 2A, A-5020 Salzburg, Austria

[2] Institute of Particle Technology, Friedrich-Alexander University Erlangen-Nürnberg, Cauerstrasse 4, 91058 Erlangen, Germany

*E-mail: gilles.bourret@plus.ac.at

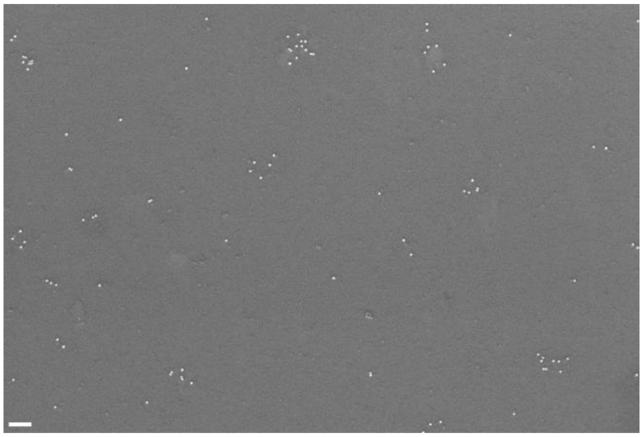

Figure S1. Secondary electron SEM image of flat Si functionalized using MPTMS only and incubated for 17 hours in AuNP solution, showing the inefficient binding of AuNPs under the used experimental conditions; scale bar: 200 nm.

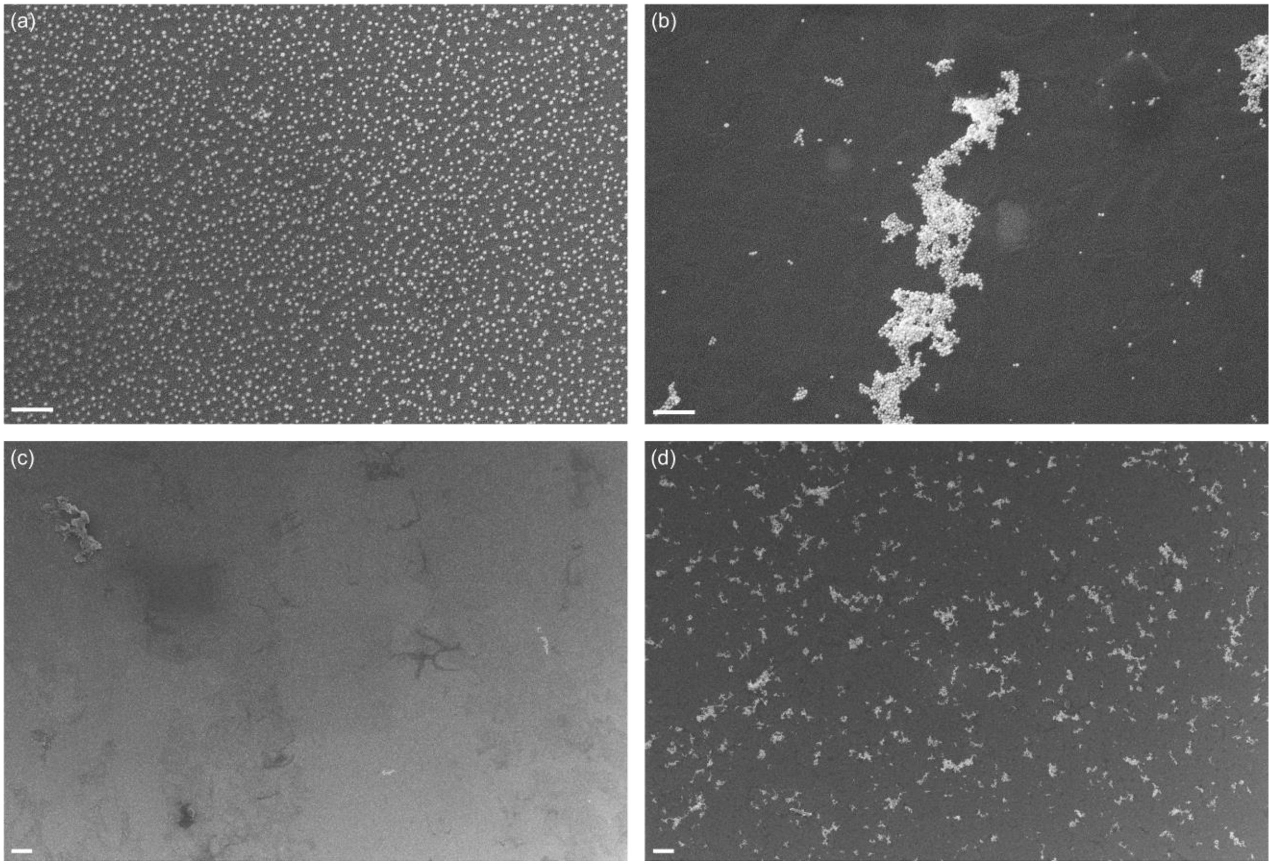

Figure S2. Secondary electron SEM images of flat Si functionalized with APTES and incubated for 4 hours in AuNP solution, (a) and (c) with, or (b) and (d) without a preliminary incubation in MilliQ water for 1 h. (a) and (b): Scale bar: 200 nm. (c) and (d): Scale bar: 2000 nm. The pre-incubation clearly leads to a more regular distribution of the AuNPs over the surface and the formation of micron-sized aggregates is avoided.

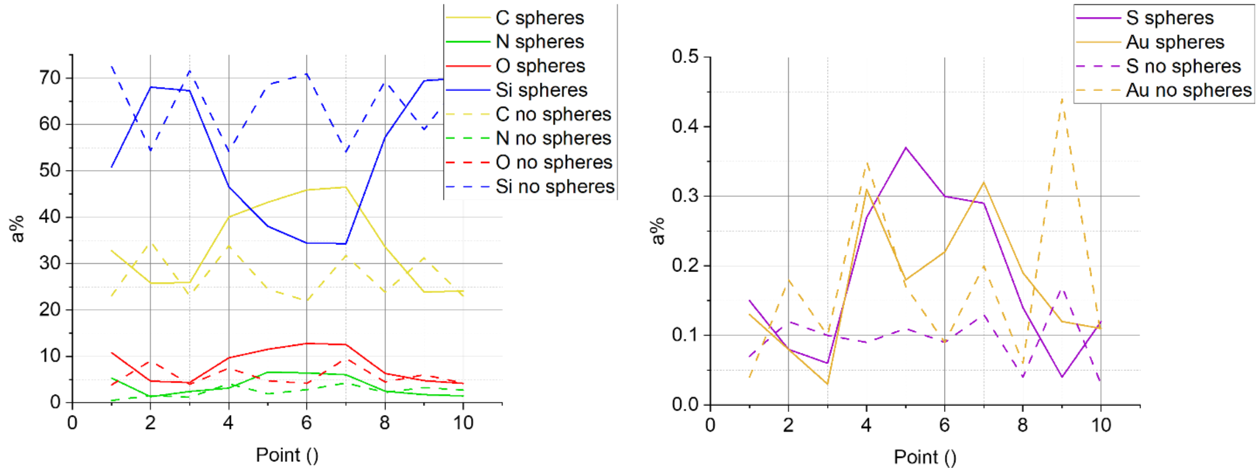

Figure S3. Results of the EDX line scans (giving the atomic percent a%) on a mixed APTES/MPTMS-SiNW array sample incubated for 4 hours in a region with (solid lines) and without (dashed lines) spherical structures. The spherical structure is located between point 3 and point 9.

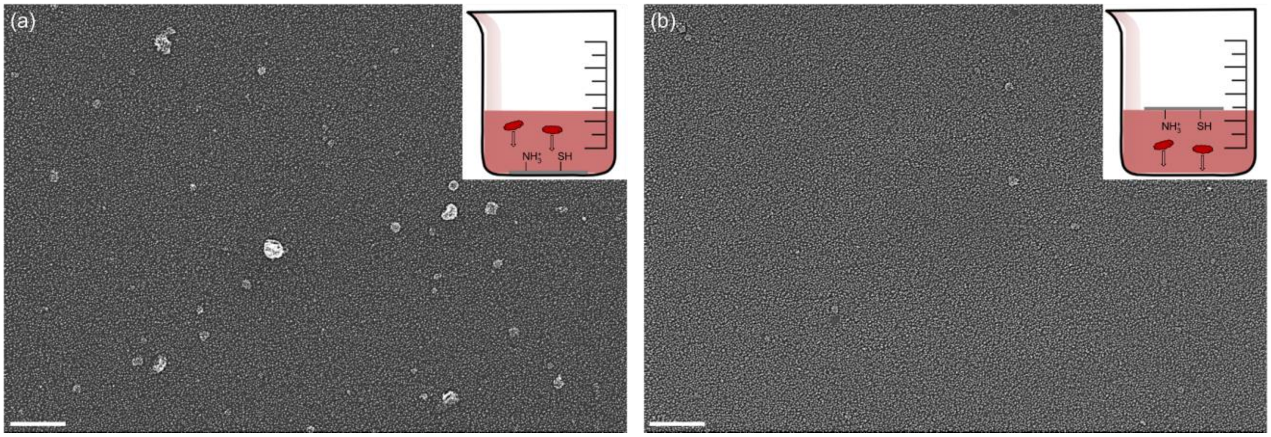

Figure S4. Secondary electron SEM images and schemes of flat Si functionalized with APTES/MPTMS and incubated for 24 hours in AuNP solution (a) lying at the bottom of the beaker containing the AuNP solution, or (b) floating on top of the AuNP solution with the silane functionalized side facing downwards. The floating approach is suited to significantly reduce the binding of spherical structures on the samples. Scale bar: 1000 nm.

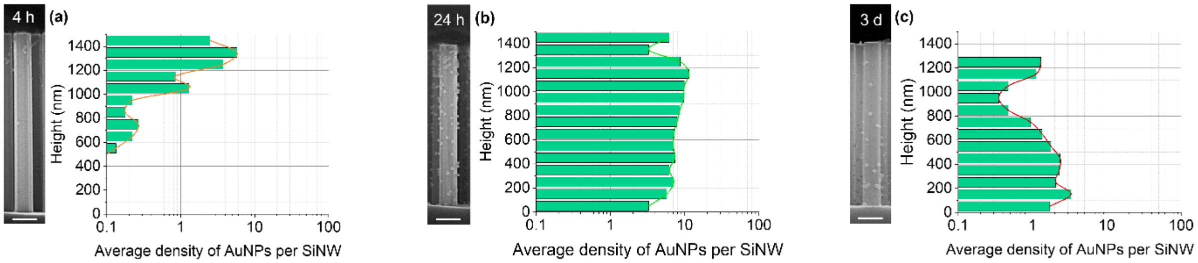

Figure S5. Spatial distribution of the AuNPs per SiNW along the nanowire long-axis utilizing an aged silane for the functionalization for three different incubation times: (a) 4 hours, (b) 24 hours, (c) 3 days, together with mixed secondary and back-scattered electron SEM images of one representative SiNW (scale bars: 200 nm).

As can be seen in **Figure S5**c, after 3 days of incubation a lower number of AuNPs was found on the SiNWs, which accumulated at the bottom of the SiNWs. These results are quite different from what we routinely observe using a fresh silane, where increasing incubation time increases AuNP density under our experimental conditions. We tentatively attribute these findings to stability issues of the silane layer prepared on the Si surface, which can degrade after few hours in aqueous solution depending on the curing conditions used to stabilize the silane layer [1-3] and after several days of storage in humid environment.[4] This could explain the desorption of the AuNPs after extended incubation times. Due to possible diffusion limited mass-transport through the nanowire arrays, the silane layer is expected to degrade faster at the top of the nanowire than at the bottom, leading to the site-selective assembly of the AuNPs at the bottom of the VA-SiNWs after extended incubation in a 1:1 mixture of AuNP solution and absolute ethanol. Overall this suggests that monolayers formed using a fresh silane are more stable than those prepared with an aged silane.

Table S1. Peak intensities and relative standard deviations for the peak at a Raman shift of ca. 1075 cm^{-1} in the baseline corrected SERS spectrum of 4-MBA, for all tested substrates.

Sample	Average peak intensity (cps)	Relative standard deviation (%)
AuNPs@APTES-flat Si 4 hours	9	99
AuNPs@APTES-flat Si 24 hours	305	22
AuNPs@APTES/MPTMS-flat Si 4 hours	25	50
AuNPs@APTES/MPTMS-flat Si 24 hours	46	40
AuNPs@APTES-SiNWs 4 hours	76	59
AuNPs@APTES-SiNWs 24 hours	1142	29
AuNPs@APTES-SiNWs 3 days	1926	37

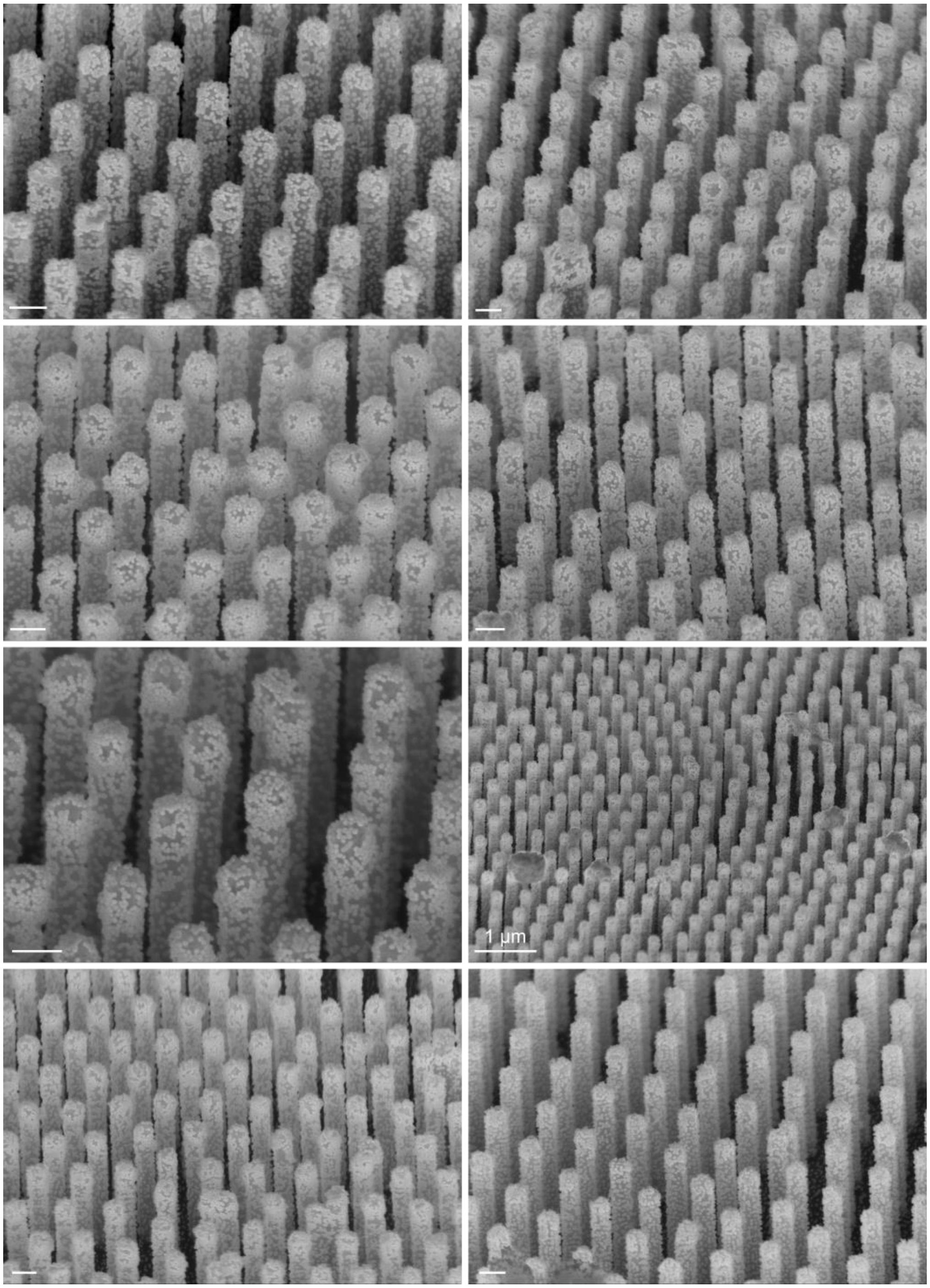

Figure S6. Additional mixed secondary and back-scattered electron SEM images in tilted view (tilt angle = 45 °) of APTES functionalized SiNWs after incubation for 3 days in a 1:1 mixture of AuNP solution and absolute ethanol. The scale bars correspond to 200 nm unless stated otherwise.

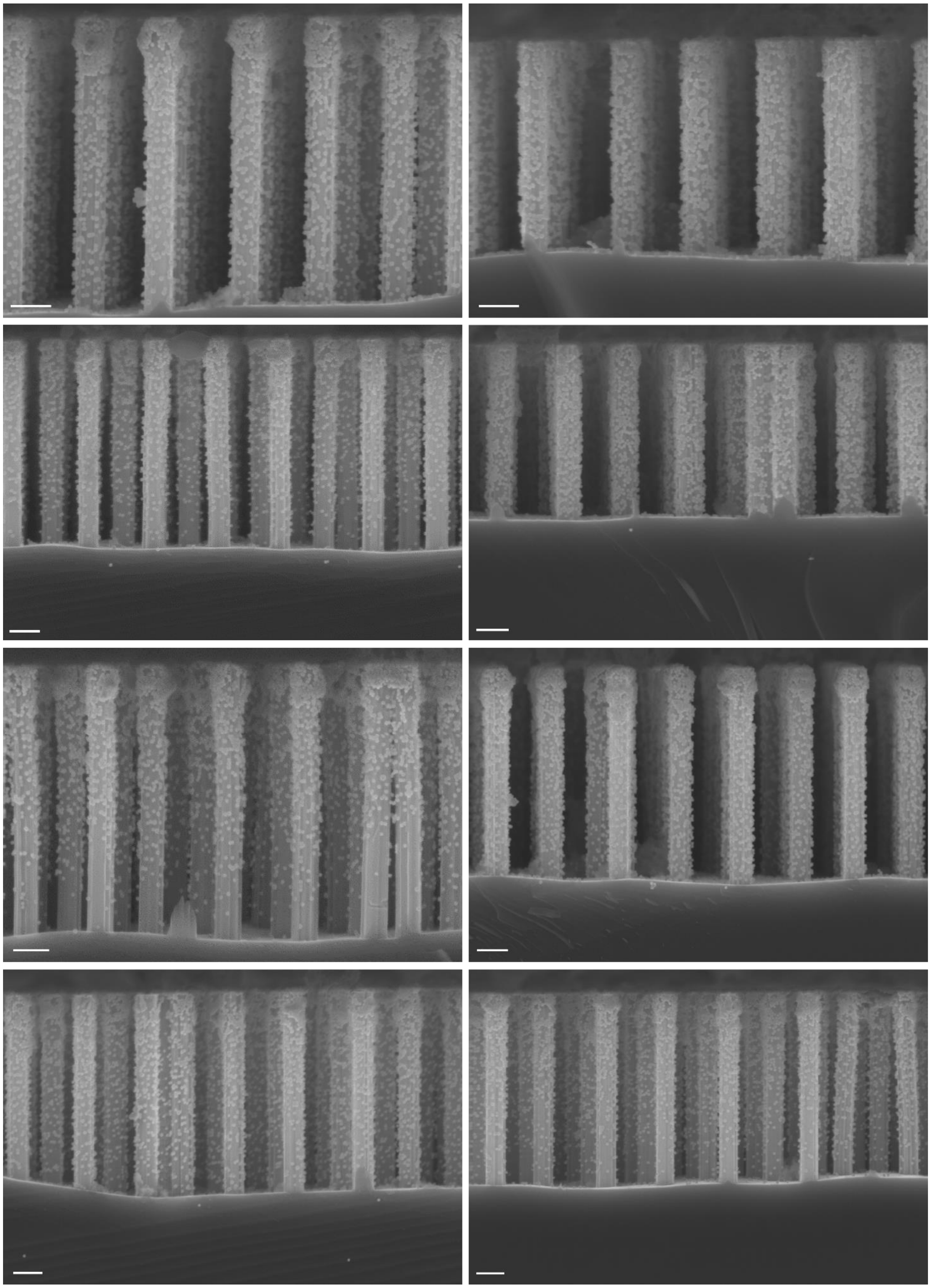

Figure S7. Additional mixed secondary and back-scattered electron SEM images in cross-sectional view of APTES functionalized SiNWs after incubation for 3 days in a 1:1 mixture of AuNP solution and absolute ethanol. The scale bars correspond to 200 nm.

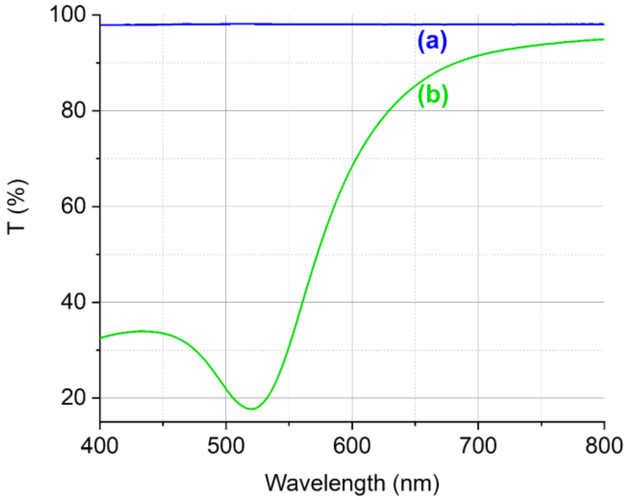

Figure S8. Original raw UV-Vis transmittance spectra of (a) water and (b) AuNP solution.

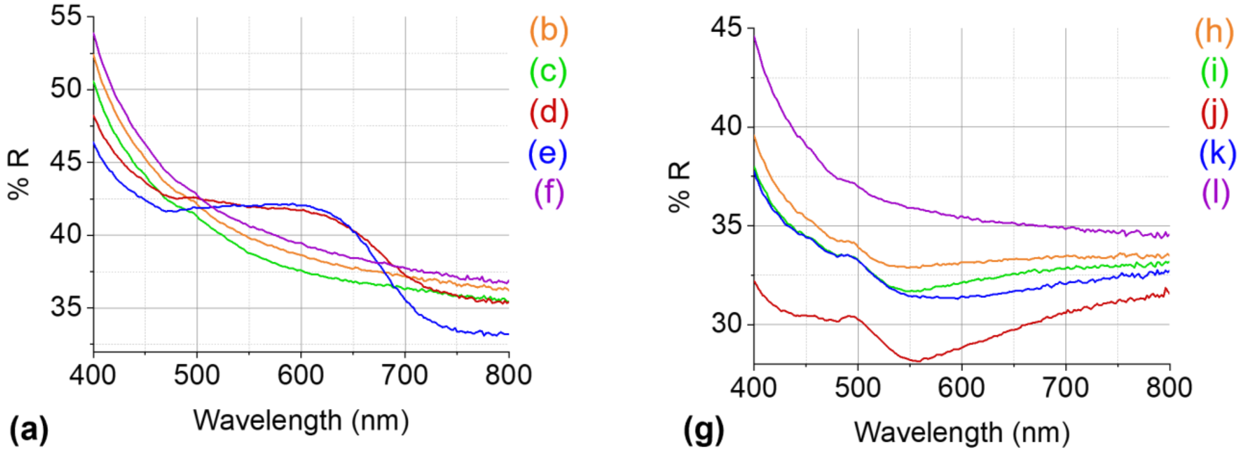

Figure S9. Original raw UV-Vis diffuse reflectance spectra of (a) APTES functionalized flat Si surfaces incubated in MilliQ water for 1 hour and in AuNP solution for (b) 1 hour, (c) 8 hours, (d) 17 hours, (e) 24 hours and (f) 0 hours (reference) and of (g) APTES/MPTMS functionalized flat Si incubated in AuNP solution for (h) 1 hour, (i) 2 hours, (j) 4 hours, (k) 24 hours and (l) 0 hours (reference).

References

1. Pasternack, R. M.; Rivillon Amy, S.; Chabal, Y. J. Attachment of 3-(aminopropyl) triethoxysilane on silicon oxide surfaces: dependence on solution temperature. *Langmuir* **2008**, *24* (22), 12963-12971.
2. Aissaoui, N.; Bergaoui, L.; Landoulsi, J.; Lambert, J. F.; Boujday, S. Silane layers on silicon surfaces: mechanism of interaction, stability, and influence on protein adsorption. *Langmuir* **2012**, *28* (1), 656-665.
3. Kim, J.; Seidler, P.; Fill, C.; Wan, L. S. Investigations of the effect of curing conditions on the structure and stability of amino-functionalized organic films on silicon substrates by Fourier transform infrared spectroscopy, ellipsometry, and fluorescence microscopy. *Surf Sci* **2008**, *602* (21), 3323-3330.
4. Lisi, F.; Carta, D.; Villanova, L.; Poli, I.; Buso, D.; Costacurta, S.; Hill, A. J.; Falcaro, P. Influence of the relative humidity on aminosilane molecular grafting properties. *J Solgel Sci Technol* **2011**, *60* (3), 246-253.
5. Michota, A.; Bukowska, J. Surface-enhanced Raman scattering (SERS) of 4-mercaptobenzoic acid on silver and gold substrates. *J Raman Spectrosc* **2003**, *34* (1), 21-25.
